# A novel sialyl Le^X^ expression score as a potential prognostic tool in colorectal cancer

**DOI:** 10.1186/1477-7819-10-95

**Published:** 2012-05-23

**Authors:** Leif Schiffmann, Fabian Schwarz, Michael Linnebacher, Friedrich Prall, Jens Pahnke, Helga Krentz, Brigitte Vollmar, Ernst Klar

**Affiliations:** 1 Department of General, Thoracic, Vascular and Transplantation Surgery, University of Rostock, Schillingallee 35, 18057, Rostock, Germany; 2 Institute of Pathology, University of Rostock, Strempelstrasse 14, 18055, Rostock, Germany; 3 Department of Neurology, Neurodegeneration Research Laboratory (NRL) and German National Dementia Center (DZNE), University of Rostock, Gehlsheimer Strasse 20, 18147, Rostock, Germany; 4 Institute for Biostatistics and Informatics in Medicine and Ageing Research, University of Rostock, Ernst-Heydemann-Strasse 8, 18057, Rostock, Germany; 5 Institute for Experimental Surgery, University of Rostock, Schillingallee 57, 18057, Rostock, Germany

**Keywords:** Colorectal cancer, prognosis, sialyl Le X, tumor staging

## Abstract

**Background:**

Treatment decisions in colorectal cancer subsequent to surgery are based mainly on the TNM system. There is a need to establish novel prognostic markers based on the molecular characterization of tumor cells. Evidence exists that sialyl Le^X^ expression is correlated with an unfavorable outcome in colorectal cancer. The aim of this study was to establish a simple sialyl Le^X^ staining score and to determine a potential correlation with the prognosis in a series of advanced colorectal carcinoma patients.

**Methods:**

In order to implement routine use of sialyl Le^X^ immunohistology, we established a new, easily reproducible score and defined a cutoff which discriminated groups with better or worse outcome, respectively. We then correlated sialyl Le^X^ expression of 215 UICC stage III and IV patients with disease-free and cancer-related survival.

**Results:**

A five-stage score could be established based on automated immunohistochemical stainings. Using a statistical model, we calculated a cutoff to discriminate between weak and strong staining positivity of sialyl Le^X^. Patients with strong positive specimens had a worse cancer-related survival (*P* = 0.004) but no difference was observed for disease-free survival (*P* = 0.352).

**Conclusions:**

These results demonstrate a strong correlation between high sialyl Le^X^-expression in colorectal carcinomas and cancer-related survival. Our highly standardized and easy-to-use staining score is suitable for routine use and hence it could be recommended to evaluate sialyl Le^X^-expression as part of the standard histopathological analysis of colorectal carcinomas and to validate the score prospectively based on a larger population.

## Background

Colorectal cancer (CRC) is one of the most important causes of cancer-related death in the Western world. In Germany, approximately 71,400 patients develop CRC per year [1]. Most cancer-related deaths are not caused by the primary cancer site, but by distant metastasis. However, patients without distant metastasis at the time of surgery (International Union Against Cancer (UICC) stages I to III) still have unsatisfying five-year survival rates between 41% and 96% [2,3].

Adjuvant chemotherapy is generally recommended for UICC III and IV tumors despite the associated toxicities [4,5]. Patients’ outcome varies widely between stages with a worse outcome from stage I to IV, but also within each stage. This is presumably due to diverse tumor phenotypes. The challenge lies in the definition of new prognostic markers for the identification of patients with a worse outcome. By identifying those patients, it would be possible to extend the indication for an adjuvant treatment to UICC stages I and II as well as for a more aggressive first-line adjuvant therapy in UICC stage III patients or palliative therapy in UICC stage IV patients.

In recent years, the terminal tetrasaccharide sialyl Le^X^ has been proposed as a promising prognostic candidate [6-8]. Its expression is increased with higher tumor stage and it has been correlated with a worse prognosis within each stage. However, so far, evaluation of sialyl Le^X^ expression has not become a standard in the histological analysis of colorectal carcinoma for diagnostic purposes or for therapeutic decision making.

Physiologically, sialyl Le^X^ is an E-selectin ligand carbohydrate structure and one of the most important blood group antigens. It is displayed on the terminus of glycolipids that are present on the cell surface. Sialyl Le^X^ is constitutively expressed on granulocytes and monocytes and mediates inflammatory extravasation of these cells [9,10]. Its capacity to permit cellular motility in conjunction with mucins explains its contribution to tumor cell spreading and metastasis [11].

We here report the development of a novel reproducible sialyl Le^X^ expression score which investigates the relationship between sialyl Le^X^ expression and prognosis in a series of patients with colorectal cancer.

## Methods

### Patients

From January 2000 to December 2004, 516 patients underwent radical surgery for CRC in the department of General, Thoracic, Vascular und Transplantation Surgery. All patients were treated according to standard treatment guidelines [12]. A preoperative anesthesiological evaluation was obtained according to the American Society of Anesthesiologists (ASA) general classification [13] to determine state of health and comorbidity. Preoperative staging involved endoscopy and biopsy, abdomen ultrasound or computer tomography and chest radiography. In case of rectal cancer, an additional endoluminal ultrasound for local staging was performed. Furthermore, patients with a locally advanced rectal cancer (≥ T3 or N+) received neoadjuvant radiochemotherapy as described previously [14]. Starting in January 2002, 5-fluoruracil (FU) treatment was changed to capecitabine.

Patients were staged according to the tumor node metastasis (TNM) system [15]. Clinical data was retrieved retrospectively. Data recorded included gender, type of admission, comorbidities, ASA-score, tumor characteristics, type of resection, morbidity and 30-day mortality. Follow-up information was recorded regarding recurrence and distant metastasis, overall survival and cancer-related survival as well as information about adjuvant therapy in early 2010. This systematic approach enabled a comprehensive data collection. The study was approved by the ethics committee of the medical faculty of Rostock University.

In total, 278 stage III and IV patients were identified. Quality assurance of collected tumor material (H & E stains) resulted in 215 tumors that were finally analyzed for sialyl Le^X^ expression.

### Immunohistochemical staining

From each paraffin-embedded tumor, sections of 4 μm were cut and mounted on polysine-covered slides. As primary antibody, the monoclonal anti-sialyl Le^X^ mouse-immunoglobulin M (IgM) (clone KM93, Kamiya Biomedical Company, Seattle, WA, USA) was used. Immunohistochemistry including deparaffinization and antigen retrieval was carried out by the fully automated Menarini Bond Max^TM^ autostainer (Menarini Diagnostics*,* Florence, Italy). After blocking endogenous peroxidase, the primary antibody (diluted 1:120 in PBS) was incubated on the sections for thirty minutes at room temperature. Sections were washed and the secondary antibody, as well as the polymer from the integrated Bond^TM^ Polymer Refine Kit (item number DS9800, Leica Microsystems, Wetzlar, Germany), were added for eight minutes each. Mixed DAB refine (Leica Microsystems) was applied for twenty minutes followed by counterstaining with hematoxylin for additional five minutes. The stainings of the control sections were constant in all runs.

### Evaluation of the sialyl Le^X^-staining

**Figure 1:**
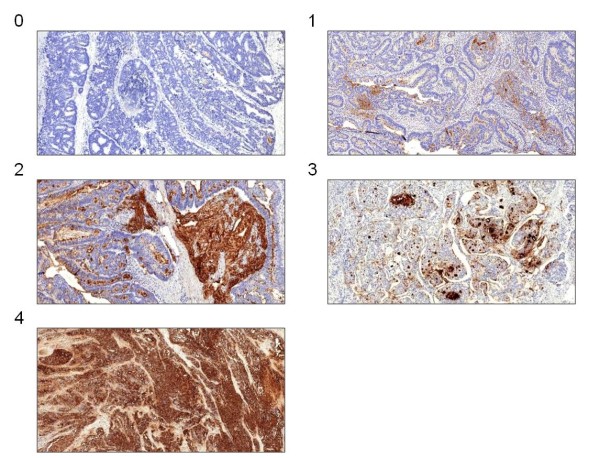
**Showcase the image sections of the five typical immunohistochemical staining patterns of sialyl Le**^**X**^**(100x magnification).**

Initially, 100 stained tumor sections were analyzed to characterize and categorize typical sialyl LeX staining patterns independently of the tumor stage. We established a five-stage score based on the amount of specifically stained tumor cells and the presence of extracellular mucus staining (Figure 1). It was not necessary to distinguish between weak and strong stained extracellular mucus in the groups with scattered or almost all stained cells. On the basis of this score, all 215 sections were classified independently by two examiners. In case of differing results, the section was reevaluated in a dialogue process.

### Statistical analysis

Statistical analysis was performed using Statistical Package for Social Science (SPSS™) version 15.0. Statistical analysis was done using Pearson’s chi-square test of Fisher’s exact test. Survival curves were calculated according to the Kaplan-Meier method. Survival curves were tested for significant differences using the log-rank test. A *P* value of <0.05 was considered as statistically significant.

The martingale residuals [16] were calculated and represented by a smoothed residual plot [17] to determine if and which cutoff value of the sialyl Le^X^-score would allow the best separation of the groups of patients with short- and long-term survival prognosis.

## Results

Patients’ characteristics as well as an overview of the therapeutic regimen of the included 215 cases with stage III and IV CRC are listed in Table 1. As expected, established prognostic markers UICC stage, T and N classification, as well as the tumor grading, showed a significant difference in cancer-related survival (data not shown).

**Table 1:** Patients’ characteristics

	Patients	n (%)
Patients	215	(100)
Mean age (years)	67.4	
Gender ratio (f/m)	1:1.26	(95:120)
Rectal cancer patients	73	(34.0)
Neoadjuvant radiochemotherapy	7	(9.6)
Colon and rectal cancer patients UICC stage III	113	(52.6)
Adjuvant (radio-) chemotherapy	89	(78.8)
Colon and rectal cancer patients UICC stage IV	102	(47.4)
Palliative (radio-) chemotherapy	62	(60.8)
Tumor location		
Right hemicolon	62	(43.7)
Transverse colon	18	(12.7)
Left hemicolon	6	(4.2)
Sigmoid colon	56	(39.4)
Rectum	73	(34.0)

**Figure 2:**
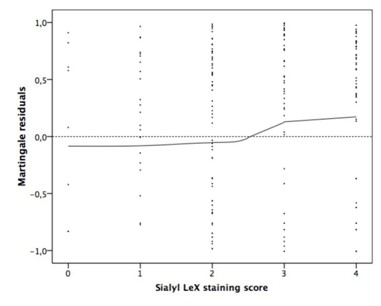
**Martingale residuals as a function of the sialyl Le**^**X**^**staining score**. Each dot represents the difference between the observed individual status and the calculated cumulative risk at the end of the observation period.

**Figure 3:**
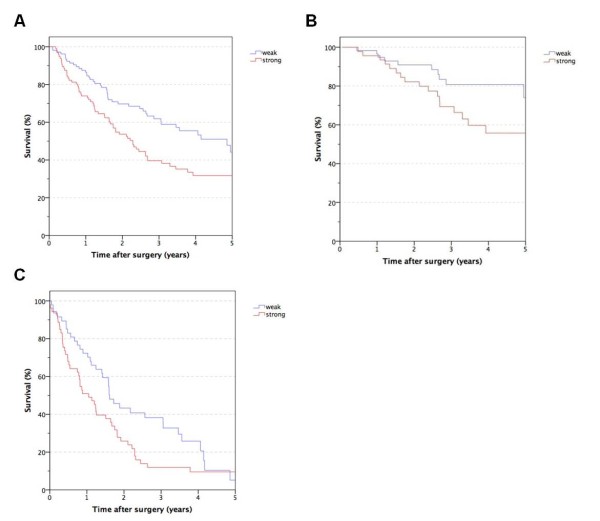
**Cancer-related survival after resection of colorectal cancer depending on sialyl Le**^**X**^**expression stages III and IV (A), stage III alone (B) and stage IV alone (C).**

Our sialyl Le^X^ evaluation score is shown in Figure 1. Basically, we distinguished between the proportion of stained cells with an extra category for extracellular stained mucus. The smoothed residual plot in Figure 2 indicates an increase in the risk of cancer-related death from score two to three. So we separated our patients into two risk groups with a staining score from zero to two (the low-risk group with weak sialyl Le^X^ expression) and three to four (the high-risk group with strong sialyl Le^X^ expression). One hundred and thirteen colorectal tumors (52.6%) were found to express sialyl Le^X^ weakly whereas 102 tumors (47.4%) showed a stronger expression. The survival curves for these two groups differ significantly and thus prove a difference in cancer-related survival (*P* = 0.004, Figure 3A). This difference persists when analyzing stage III (*P* = 0.033, Figure 3B) and stage IV patients separately (*P* = 0.044, Figure 3 C). However, there was no difference in disease-free survival between the two patient groups with weak and strong sialyl Le^X^ expression (*P* = 0.352). Likewise, the increase of sialyl Le^X^ expression in carcinomas with UICC stage III (43.4%) and IV (52.0%) was not statistically significant in our patient population (*P* = 0.131). Distribution of age, gender, common histopathological features and tumor localization were comparable between weak and strong sialyl Le^X^-expressing carcinomas (Table 2).

**Table 2:** **Clinical and histopathological criteria stratified by sialyl Le**^**X**^**expression**

	Weak sialyl Le^X^ expression n (%)		Strong sialyl Le^X^ expression n (%)	
Patients	113	(52.6)	102	(47.4)
Mean age (years)	67.2		67.6	
Gender ratio (f/m)	1:1.57	(44:69)	1:1	(51:51)
Infiltration (pT)				
T1	2	(1.8)	0	(0.0)
T2	13	(11.5)	6	(5.9)
T3	54	(47.8)	42	(41.2)
T4	44	(38.9)	54	(52.9)
Lymph node metastasis (pN)				
N0	10	(8.8)	9	(8.8)
N1	61	(54.0)	39	(38.2)
N2	42	(37.2)	54	(52.9)
Metastasis				
M0	64	(56.6)	49	(48.0)
M1	49	(43.4)	53	(52.0)
Grading				
G1	18	(15.9)	26	(25.5)
G2	79	(69.9)	70	(68.6)
G3	16	(14.2)	6	(5.9)
Localization				
Colon	70	(61.9)	72	(70.6)
Rectum	43	(38.1)	30	(29.4)

## Discussion

Long-term survival of CRC patients is limited by metastasis rather than by the primary tumor itself. So far, an adjuvant treatment is administrated based on prognosis determined by the UICC stage. However, the TNM system does not allow stratification of certain subgroups of patients in whom the outcome is predominantly determined by tumor cell-specific molecular characteristics. This underlines the need for additional prognostic markers.

The tetrasaccharide carbohydrate sialyl Le^X^ appears to be a promising candidate marker to reflect tumor aggressiveness. Several groups proposed that immunohistochemical assessment of sialyl Le^X^ expression is of prognostic relevance [6-8].

In the present work, we analyzed tumor sections of 215 colorectal carcinoma patients with a documented follow-up. Because the correlation of stronger sialyl Le^X^ expression with higher UICC stages has been shown previously [8] and shorter survival times make statistical evaluation more feasible, we analyzed only stage III and IV patients. Our patient cohort is representative since there was a correlation with survival and the well-established prognostic markers T, N and G as well as the UICC stages.

Immunohistochemistry is ideal to depict sialyl Le^X^ expression since PCR is not the method of choice to analyze carbohydrates and in Western blot the sialyl Le^X^ signals may be distributed from different cellular compartments. The differentiation between staining signals generated by the extracellular mucus and the cell membrane is a main challenge in evaluating sialyl Le^x^ expression. Only the latter is thought to be basically linked with the extravasation of tumor cells [18]. So far, this discrimination has not been achieved [6-8,19]. To limit variance in staining, we chose a fully automated processing of the slides.

Due to the changing properties of the specimens, a score based on staining intensity as well as the percentage of stained cells, as suggested by Grabowski *et al*. [8], might not be reproducible even when using automated staining systems. Staining intensity especially could vary by minor changes in concentrations, staining time or the age of the staining components. The group of Nakamori [6] distinguished between negative- and positive-stained cells with a cutoff at 5%. Nakagoe *et al*. [7] categorized four groups with cutoffs at 5%, 40% and 80%. These approaches were not widely accepted, possibly due to the fact that natural staining patterns were not considered sufficient. Variations as well as difficulties in interpretation of staining might have limited the acceptance of the previous techniques. Therefore, we defined a score that allows an easy categorization independent from staining intensities. It takes into account the proportion of positive tumor cells and the staining of the extracellular mucus. Principally, it appears possible to merge the categories of sporadic positively stained cells with and without extracellular mucus (score 1 and 2) to one category.

As a statistical model to distinguish between groups with higher and lower risk of tumor-related death, we used the martingale residuals [16,17,20]. The best cutoff was detected between a score of two and three, by which we could confirm the correlation between sialyl Le^X^ expression and tumor-related survival found by others [6-8]. However, our findings differ in some points. First, unlike Grabowski and coworkers [8], we observed a significant difference in survival in UICC stage III as well as IV. This finding raises some skepticism to their hypothesis that there is no influence of sialyl Le^X^ expression in UICC stage IV patients due to pre-existing metastases. The difference in survival observed by us may partly be explained by the fact that, unlike multiple metastases, a single metastasis or limited metastatic disease do not necessarily limit patients’ long-term survival [21,22]. Surgical approaches have become much more aggressive with a survival benefit for the patients. Second, we observed no significant difference in disease-free survival in stage III patients between the two groups with low and high sialyl Le^X^ expression. Previous studies did not focus on this question [6,8]. Thus, future studies with prolonged follow-up time and increased patient numbers might identify significant differences.

Comparable to literature data, we found no correlation between sialyl Le^X^ expression and other histopathological markers like TNM or grading [6-8]. The increase of sialyl Le^X^ expression from carcinomas of UICC stage III (43.4%) to stage IV (52.0%) was not significant in the presented study. This fact might be a problem with the size of the study population and might become significant in a larger study cohort.

## Conclusions

In conclusion, our data strongly support previous findings that increased sialyl Le^X^ expression is predictive for a worse outcome. We developed an easy scoring system feasible to distinguish between weak and strong sialyl Le^X^ expression. A significant difference was observed for cancer-related survival but not for disease-free survival. In consequence, we recommend sialyl Le^X^ immunohistochemistry to be further evaluated on a routine basis. When taking into account previous studies on sialyl Le^X^ expression, it seems promising to use it as an independent prognostic marker to identify subgroups of patients with a worse prognosis.

## Competing interests

The authors declare that they have no competing interests.

## Authors’ contributions

LS and FS conceived and coordinated the study, collected patients’ data, participated in the analysis of the specimens and statistical analysis. LS drafted the manuscript. FP identified the paraffin blocks and participated in preparing the manuscript. JP established the automated staining and participated in developing the new score. HK participated in the statistical analysis. BV participated in conceiving and coordinating the study and preparing the manuscript. ML and EK participated in preparing and drafting the manuscript. All authors read and approved the final manuscript.
